# Forecasting demand for blood products: Towards inventory management of a perishable product

**DOI:** 10.6026/973206300200020

**Published:** 2024-01-31

**Authors:** Sanjay Kumar Thakur, Anil Kumar Sinha, Dinesh Kumar Negi, Sompal Singh

**Affiliations:** 1 P.G. Department of Zoology, Veer Kunwar Singh University, Ara, Bihar-802301, India; 2 Department of Regional Blood Transfusion Centre and Department of Pathology, Hindu Rao Hospital and NDM Medical College, Delhi-110007, India

**Keywords:** Inventory management, Demand forecasting, Blood donation, Blood issue

## Abstract

Forecasting consumption of blood products can reduce their order frequency by 60% and inventory level by 40%. This also prevents shortage by balancing demand and supply. The study aimed to establish a "Simple Average with Mean Annual Increment" (SAMAI) method of time series forecasting and to compare its results with those of ARIMA, ratio to trend, and simple average to forecast demand of blood products. Monthly demand data of blood component from January 2017 to December 2022 (data set I) was used for creating a forecasting model. To avoid the effect of COVID19 pandemic instead of actual data of year 2020 and 2021, average monthly values of previous three years were used (data set II). The data from January to July 2023 were used as testing data set. To assess the fitness of model MAPE (Mean Absolute Percentage Error) was used. By SAMAI method MAPE were 18.82%, 13.392%, 14.516% and 27.637% respectively for of blood donation, blood issue, RDP issue and FFP issue for data set I. By Simple Average method MAPE were 20.05%, 12.09%, 29.06% and 34.85%, respectably. By Ratio-to-Trend method MAPE were 21.08%, 21.65%, 25.62% and 39.95% respectively. By SARIMA method MAPE were 12.99%, 19.59%, 37.15% and 31.94% respectively. The average MAPE was lower in data set II by all tested method and overall MAPE was lower by SAMAI method. The SAMAI method is simple and easy to perform. It can be used in the forecasting of blood components demand in medical institution without knowledge of advanced statistics.

## Background:

Global healthcare systems face significant challenges in improving supply chain performance due to the complexity of their supply chains, which are closely linked to human health. The medical community is focusing on strengthening supply networks to reduce risks and waste while maintaining customer service standards [1,2]. The main challenges in the health supply chain include demand uncertainty, inventory management, expiration, and a lack of resources [2,3,4]. Planning blood collection and distribution is crucial for hospitals and healthcare facilities, especially in the production and distribution of various blood components. Forecasting blood consumption ensures balance between demand and supply, preventing inventory shortages or oversupply [4]. This allows for rational resource allocation and clinical need coordination. However, managing the supply chain of delicate blood products like platelets having a limited shelf life is challenging due to wastage of blood products [5]. Accurate projections of blood demand are essential for making wise choices and managing blood supplies. Gathering data over years helps determine demand characteristics and forecast future demand [4,6].

Currently, a few haphazard methods have been used to estimate blood demand in basic deterministic models. These methods include using demographic information for age distribution, age-specific disease prevalence, donor recruitment rates, donation frequency, RBC transfusion data, and/or blood requirements based on various disease indications, among other things [7, 8,9]. However, these models haven't been able to correctly forecast how clinical transfusion procedures would alter [7-9]. Use of a time-series methods have shown potential for high accuracy in forecasting demand for RBC transfusions [4, 8]. Time-series models have been used in various domains such as public health and biological data aspects [10], brain studies [11], drug usage [12], gene networks [13], traffic safety [14], prediction of COVID19 outbreak [15], prediction of RBC demand [8,16] and so on. Li *et al.* [6] proposed a decision integration technique for short-term demand forecasting that integrates a hybrid demand forecasting model based on statistical time-series modelling, machine learning, and operations research. The machine learning models are useful for prediction of patients with specific diseases, such as trauma [17], preoperative [18], mitral valve [19], liver transplant surgery [20], disease burden [21] and blood demand [22], The results demonstrated that the suggested method can reduce order frequency by 60% and inventory levels by 40%, potentially lowering shortages and waste from expiration [4].

Addis *et al.* [3] emphasize the importance of considering a solution's robustness, efficacy, cost, and ease of application before implementing a method requiring specialist knowledge. Medical staffs with heavy workloads often have limited time to adopt new methodologies or techniques, especially in laborious analytics. Health workers are typically untrained in complex statistical analysis and economic approaches, suggesting that ordinary practitioners should have little trouble understanding and implementing statistical approaches for scheduling and prediction, necessitating greater investment in new technologies [4]. Therefore, it is of interest to establish a "Simple Average with Mean Annual Increment" (SAMAI) method of time series forecasting and to compare its results with those of ARIMA, ratio to trend, and simple average to forecast demand of blood products.

## Materials and Methods:

## Ethical Considerations:

Present study was approved by the Institutional Ethical Committee of Hindu Rao Hospital and NDMC Medical College, Delhi by the approval number- F. No: IEC/NDMC/2021/69. For present study, the data of routine blood donations, blood issue, random donor platelets (RDP) issue and fresh frozen plasma (FFP) issue was collected from inventory registers of Regional Blood Transfusion Centre, Delhi.

## Prediction by simple average with mean annual increment method (SAMAI):

This technique assumes the presence of trend and seasonality and absence of cyclical changes. The method consists of the following steps.

[1] The data are arranged year-wise on monthly basis.

[2] The monthly average is calculated for each month, by dividing the total of each month by the number of months added.

[3] The average of monthly average (grand average) is calculated by dividing the total of monthly average by number of months in year (12).

[4] Every month's seasonal index (SI) is calculated using the formula below:

See PDF

[5] For each year, the total of annual values is calculated and called annual total.

[6] Each next year annual total is divided by annual total of previous year and annual increment ratio is obtained.

[7] Grand total of annual increment ratio is obtained by adding them.

[8] The mean of annual increment ratio is obtained by dividing number of annual increment ratio.

[9] Each monthly average is multiplied by mean of annual increment ratio for the prediction of next year monthly value or by using formula below:

See PDF

## Prediction by simple average method:

This technique is based on the additive modal of the time series. This technique assumes the absence of trend and cyclical changes [23]. The method consists of the following steps.

[1] Step 1 to 4 is same as SAMAI method.

Every month's predictions are calculated using the formula below:

See PDF

## Prediction by Ratio to trend method:

In this method, the trend is computed using the least squares method [23]. The steps are as follows:

[1] Every year, the average of the actual values for each year is determined. Based on all such averages, the values of trend of various years are obtained by the method of least squares. These represent the trend values for the corresponding year's midpoints. Using the change in trend per annum and the change in trend per season (and the change in trend per half a season when required), the trend values of all the seasons are calculated.

[2] Ratio-to-Trend of each season is obtained by

See PDF

[3] Such ratios are in percentages. They are tabulated season wise in chronological order.

[4] The total and the average of each season are found

[5] The average of those seasonal averages is found and called "Grand Average".

See PDF

To obtain the seasonal indices, multiply the seasonal averages by the correction factor.

## Model construction for prediction by ARIMA:

ARIMA model:

The AR and MA model can be stated as described elsewhere [4,8, 24] and below.

Autoregressive model (AR) Yt = α0 + α1at-1 + α2at-2+...+ αpap-1+ ε t

Moving average model (MA) Yt = εt + β1εt -1 + β2εt -2+...+ βqε t -q

Were, α1, α2 ... parameters of AR, β1, β ... parameters of MA, α0 is constant, εtis a error term at time t, p is order of AR and q is order of MA.

A combination of the AR (p) and the MA (q) terms give ARMA (p, q). Hence, we got the following ARMA equation:

Yt= α0 + α1at-1 + α2at-2+...+ αpap-1 + β1ε t -1 + β2εt -2 +...+ βqε t -q+ εt The combination of non-parametric differencing (d) and integration (I) with a parametric ARMA process give ARIMA (p, d, q) model. Where "d" represents the number of differencing operations and the "I" represents this time-series integration process in the ARIMA acronym. An ARIMA model is a model where the series of time was subtracted at least once in order to make it stationary [4,8,24].

## Seasonal ARIMA model:

The seasonal ARIMA model incorporates both non-seasonal and seasonal factors in a multiplicative model. The shorthand notation for the model is ARIMA (p, d, q) x (P, D, Q) S

With p = non-seasonal AR order, d = non-seasonal differencing, q = non-seasonal MA order, P = seasonal AR order, D=seasonal differencing, Q = seasonal MA order, and S = time span of repeating seasonal pattern.

## Econometric tests and procedures:

The Augmented Dickey-Fuller (ADF) test is used to determine data stationarity in the ARIMA model. The null hypothesis suggests no stationary, but rejecting it confirms data stationary [25]. The Box-Jenkins technique assumes data normality, while the Jarque-Bera test assesses skewness and kurtosis values.

## Model identification:

## ACF and PACF:

Autocorrelation plots, including the autocorrelation function (ACF) and partial autocorrelation function (PACF), aid in determining the parameter order of an ARIMA model, determining the differencing requirement (d) and the order of AR(p) and MA(q) parameters [4].

## AIC, BIC, LR:

The stationary test focuses on the data's autoregressive representation, using Akaike Information Criterion (AIC) and Bayesian Information Criterion (BIC) to select the best-fit model. The Hamilton-described that log Likelihood Ratio generates AIC and BIC and the model with the highest AIC/BIC function and/or lowest error selected [4, 8,24].

## Prediction by ARIMA method:

The Auto-Regressive-Integrated-Moving Average (ARIMA) model was performed using an open-source Windows statistical software package, Python version 3.0 (Python, Inc., Chicago, Illinois) and Jupyter Notebook version 6.0.3 (Python, Inc.).The best model was identified using "pmdarima" python package. Seasonal decomposition of time series data was don. The model parameters (p, d, q), (P, D, Q), AIC/BIC and model coefficients were obtained. The steps are as follows:

[1] Import libraries.

[2] Load dataset.

[3] Test for data stationary.

[4] Seasonal decomposition of dataset.

[5] Prepare train and test dataset.

[6] Plot the Auto Correlation Charts.

[7] Create SARIMA model using auto ARIMA.

[8] Plot and print Forecast.

[9] Compute accuracy.

## Evaluation of Forecasting Models:

The Mean Absolute Percentage Error (MAPE) is utilized to evaluate model performance and technique accuracy by providing a percentage-based relative value of predicting errors [4,8, 24].

See PDF

Where, n= number of times the summation iteration happens, At = actual value, Ft=forecast value

## Data collection and statistical analysis:

**Figure 1:**
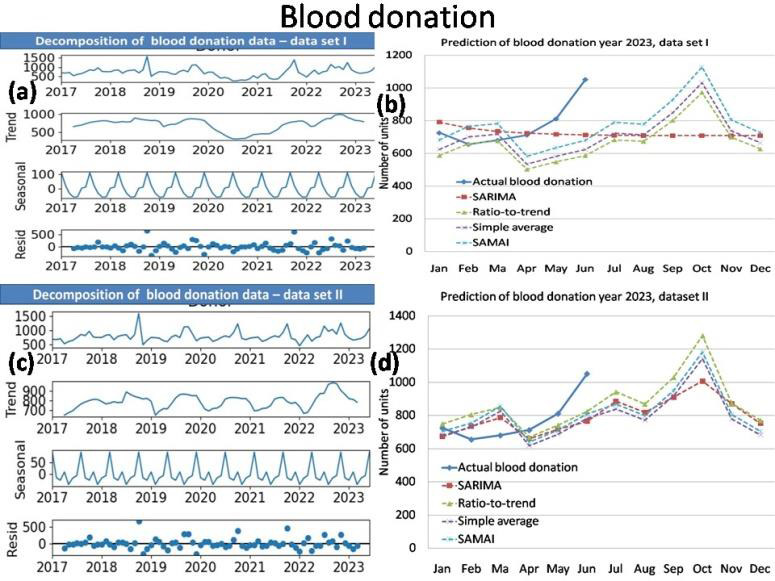
Decomposition plot (a) and Prediction of blood donation (b) year 2023 of data set I with Decomposition plot (c) and Prediction of blood donation (d) year 2023 of data set II.

**Figure 2:**
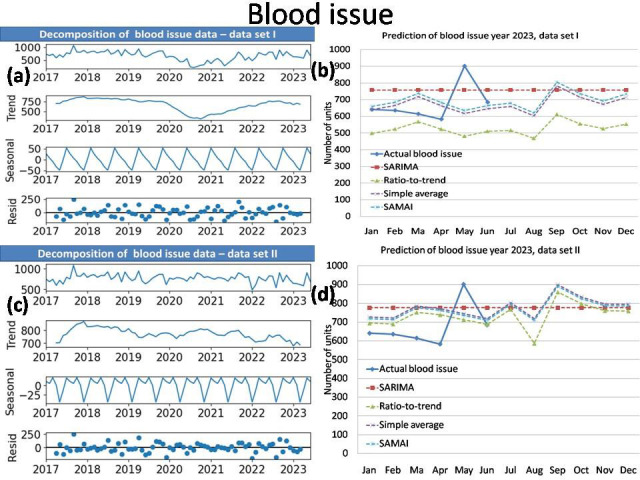
Decomposition plot (a) and Prediction of blood issue (b) year 2023 of data set I with Decomposition plot (c) and Prediction of blood issue (d) year 2023 of data set II.

**Figure 3:**
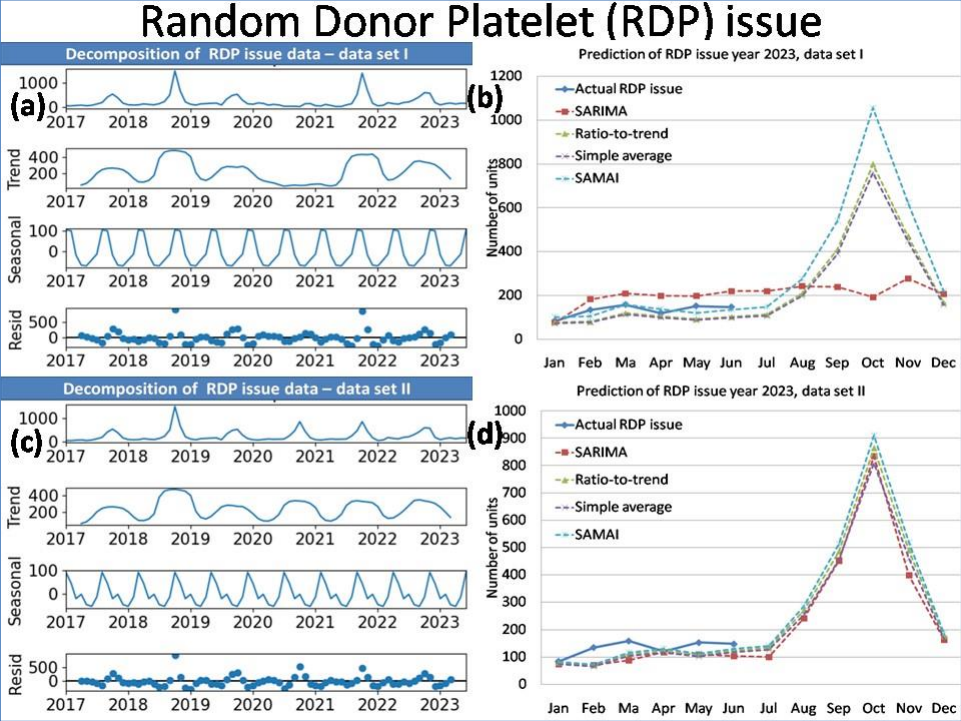
Decomposition plot (a) and Prediction of RDP issue year 2023 (b) of data set I with Decomposition plot (c) and Prediction of RDP issue year (d) 2023 of data set II.

**Figure 4:**
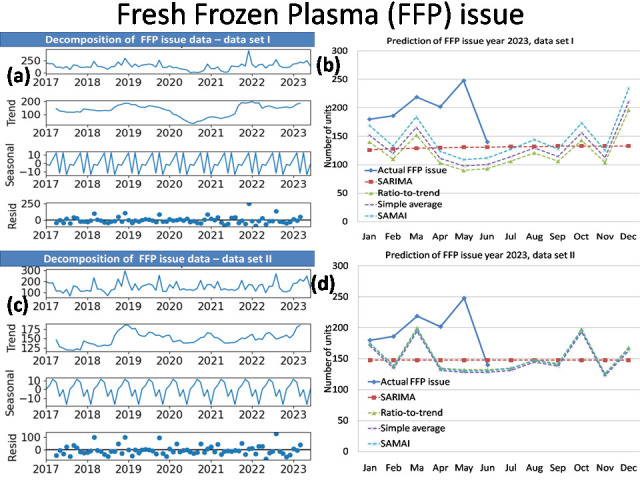
Decomposition plot (a) and Prediction of FFP issue year 2023(b) of data set I with Decomposition plot (c) and Prediction of FFP issue year (d) 2023 of data set II.

Monthly data with an annual frequency of 12 from January 2017 to July 2023 were collected and entered into the Microsoft Excel sheet for Time series analysis. During the year 2020 and 2021due to COVID19 pandemic, notable decline in blood transfusion services was observed. For better prediction and comparison of time series analysis two sets of data were prepared, data set I is the actual data whereas for data set II instead of actual data of year 2020 to 2021, average monthly values of previous three years were used. The study data from January 2017 to December 2022 was used as training data set to identify the trend and seasonal pattern. The data from January 2023 to July 2023 were used as testing data set. To assess the fitness of model MAPE (Mean Absolute Percentage Error) was used. Study results were presented in the form of Table 1,Table 2, Table 3,Table 4 and Figure 1, Figure 2,Figure 3,Figure 4.

## Results:

The time series analysis results of simple average with mean annual increment (SAMAI) and simple mean method are presented in Table 1 and Table 2, ratio to trend in Table 2 and Table 3, Auto-ARIMA in Table 2 and Table 4. Trend and MAPE of predictions obtained by ARIMA, Ratio-to-Trend, Simple Average and Simple average with mean annual increment (SAMAI) methods of Blood donation, Blood issue, RDP issue and FFP issue of data set I and II are presented together in Table 2. The decomposition of data (data series, trend, seasonal and residual) and prediction of Blood donation, Blood issue, RDP issue and FFP issue for data sets I and II by SAMAI, Simple Average, Ratio-to-Trend and ARIMA methods are presented together in Figure 1, Figure 2,Figure 3,Figure 4. The actual date with predications by simple average with mean annual increment (SAMAI), simple mean, ratio-to-trend and ARIMA methods are presented for blood donation data, blood issue, RDP issue and FFP issue Figure 1, Figure 2,Figure 3,Figure 4, respectively.

## Prediction by simple average with mean annual increment (SAMAI) method:

The seasonal index (S.I.) of Simple Average method for blood donation, blood issue and RDP issue were higher in the month of October, September and October respectively for both the data set I and II (Table 1). For FFP issue, S.I. was higher in the month of December for data set I and in the month of October for data set II. The grand yearly average for blood donation, blood issue RDP issue and FFP issue were 708.0278, 674.5833, 211.6 and 128.8833 for data set I and 788.5972, 776.0093, 234.4167and 148.5 for data set II respectively. The mean annual increment ratio for blood donation, blood issue RDP issue and FFP issue were 1.092, 1.029, 1.388 and 1.111 for data set I and 1.034, 0.989, 1.127 and 1.019 for data set II, respectively. The MAPE of Simple Average with Mean annual Increment (SAMAI) method for blood donation, blood issue RDP issue and FFP issue were 18.82%, 13.392%, 14.516% and 27.637% for data set I and 14.88%, 17.231%, 19.641% and 21.112% for data set II, respectively (Table 2).

## Prediction by simple average method:

The seasonal indices (S.I.) and grand average of Simple Average method were same as that of SAMAI method (Table 1). The MAPE of Simple Average method for blood donation, blood issue RDP issue and FFP issue were 20.05%, 12.09%, 29.06%and 34.85% for data set I and 15.49%, 18.18%, 26.08% and 22.59%for data set II respectively (Table 2).

## Prediction by ratio-to-trend method:

The seasonal index (S.I.) of Ratio-to-Trend method for blood donation, blood issue and RDP issue were higher in the month of October, September and October respectively for both the data set I and II (Table 3). For FFP issue S.I. was higher in the month of December for data set I and in the month of March for data set II. The intercept of Ratio-to-Trend method for blood donation, blood issue RDP issue and FFP issue were 704.40, 653.73, 219.22 and 130.65 for data set I and 795.68, 771.19, 238.87 and 149.00 for data set II, respectively. The slope of Ratio-to-Trend method for blood donation, blood issue RDP issue and FFP issue were -7025, -41.71, 2.93 and -2.86 for data set I and 14.17, -9.63, 4.22 and 1.15 for data set II, respectively. The MAPE of Ratio-to-Trend method for blood donation, blood issue RDP issue and FFP issue were 21.08%, 21.65%, 25.62% and 39.95% for data set I and 14.51%, 14.52%, 22.36% and 20.49% for data set II respectively (Table 2).

## Prediction by SARIMA method:

The Augmented Dickey-Fuller (ADF) test for data sets I and II was performed for the stationarity of the data. The p value of the ADF test of data set I for blood donation was 0.0002, the blood issue was 0.1424, the RDP issue was 0.0000 and the FFP issue was 0.0025. The p value of the ADF test of data set II for blood donation was 0.818, the blood issue was 0.0000, the RDP issue was 0.262271and the FFP issue was 0.000018. The data from January 2017 to December 2022 was used as training data and the data from January to June 2023 was used as testing data. The best models selected by seasonal auto-ARIMA for blood donation, blood issue, RDP issue and FFP issue and other model details are shown in Table 4. ACF and PACF graph were also plotted. The models obtained by auto-ARIMA were used to predict the next 12 months (January 2023 to December 2023). The mean absolute percentage error (MAPE) of testing and predicted data (January 2023 to June 2023) of data set I for blood donation, blood issue, RDP issue and FFP issue was 12.99%, 19.59%, 37.15% and 31.94% respectively and for data set II 13.51%, 21.60%, 27.35% and 23.78% respectively (Table 2).

## Discussion:

The Seasons of India are majorly classified as Spring (February to March), Summer (March to May), Monsoon (June to September) Autumn (October to November) and Winter seasons (December to February). Climate change is expected to cause numerous health issues in developing nations, including vector-borne and water-borne diseases like malaria, cholera, dengue and chikungunya [26]. Our time series analysis result shows our blood transfusion services has seasonality and trend. Our result shows increased blood donation, blood issue and FFP issue as first peak between Spring and Summer Season (March) and second peak during late of Monsoon and Autumn Season (October). The RDP issue shows one peak during late of Monsoon and Autumn Season (October).The Winter Season show comparatively decreased demand.

The authors Ben Elmir W *et al.* [4] found that time series prediction techniques particularly Exponential Smoothing Models (ESM) and Autoregressive Integrated Moving Average models (ARIMA), outperformed machine learning systems. The ARIMA model extracts trend and periodic information for future predictions and SARIMA model, which is a combination of multiple time series models, effectively predicts demand for therapeutic red blood cells based on seasonal cycles [4,8]. Our study results of auto SARIMA, Ratio-to-Trend and Simple average with mean annual increment (SAMAI) methods, shows increasing trend in blood donation, RDP issue and FFP issue.

Our results show, the prediction by auto SARIMA of data set I have seasonality only in Platelet issue whereas data set II has seasonality in blood donation and RDP issue. This is due to the fact that SARIMA model is based on the statistical analysis of past data to establish a model and highlights the time series and does not take into account the influence of other factors. This is due to the fact that, our data set I that represents actual data which has effects of COVID19 pandemic as decline in the blood transfusion services [27]. The data set II has average monthly values of previous three years data for year 2020 and 2021. On the other hand, the other time series methods used in this study; Ratio-to-Trend to method, Simple average method and Simple average with mean annual increment (SAMAI) shows seasonality in blood donation, blood issue, and FFP issue with two peaks in the month of March and October (Figure 1,Figure 2, Figure 3,Figure 4) both in data set I and II. In RDP issue with one peak in the month of October (Figure 1,Figure 2, Figure 3,Figure 4) both in data set I and II.

Our result shows various environmental (macro) conditions can only remain stable for a certain period of time. It may have a forecast error defect, if there were major changes, such as the outbreak of COVID19 pandemic during the year 2020 and 2021 that cause notable decline in blood transfusion services.

Therefore, continuous modify or refit the model according to the actual situation is useful to improve the prediction accuracy and ensure the fitting effect of the model. It can provide a basis for the clinical formulation of blood use plans in a timely and accurate manner.

The optimal model selected by auto SARIMA to forecast blood donation, blood issue, RDP issue and FFP issue were different (Table 1) and there is no one model that will work perfectly for all. The study results show the MAPE of the forecasted and actual values was comparatively lower in prediction of data set II compared to data set I. In overall MAPE was lowest in the prediction of SAMAI method compared to ARIMA, Ratio-to-Trend and Simple Average methods. The medical staffs with heavy work load are typically untrained in complex statistical analysis and implementing the statistical models, require specialist knowledge [3,4]. The SAMAI method is simple and easy to perform. It can be used in the forecasting of blood components demand in medical institution without advanced statistical knowledge.

## Conclusion:

Due to decline in blood transfusion services during COVID19 pandemic time series forecasting was effected. This work proposes a simple approach to predict blood demand and supply, balancing collection and distribution through effective inventory management. The SAMAI method is simple and easy to perform. It can be used in the forecasting of blood components demand in medical institution without knowledge of advanced statistics.

## Funding:

This research did not receive any specific grant from funding agencies in the public, commercial, or not-for-profit sector.

## Author contributions:

Sanjay K Thakur, Anil K Sinha, Dinesh K Negi and Sompal Singh designed the study. Sanjay K Thakur conducted literature searches, data collection, analysis, and manuscript preparation. All authors participated in data analysis, interpretation, and manuscript preparation, contributing equally to the final version's preparation and critical review.

## Figures and Tables

**Table 1:** Seasonal index and grand average of Simple average with mean annual increment (SAMAI) and Simple average method for Blood donation, Blood issue, RDP issue and FFP issue for data set I and II.

	**Blood Donation**		**Blood issue**		**RDP issue**		**FFP issue**	
**Months**	**Data set I**	**Data set II**	**Data set I**	**Data set II**	**Data set I**	**Data set II**	**Data set I**	**Data set II**
January	88.13213	86.34178	94.9475	93.62717	33.60122	30.99665	115.0789	115.0556
February	98.86617	92.59057	98.70291	92.87546	35.13203	27.24233	90.47321	90.72702
March	101.1495	104.8486	106.3373	101.266	52.35362	43.38593	125.4259	130.7757
April	75.30307	78.46563	98.33231	99.41175	45.84768	48.57159	84.41643	88.63102
May	82.106	87.28579	91.41446	95.93957	40.26024	42.56467	74.06942	86.42273
June	87.94382	99.21978	95.71341	92.57478	45.00574	49.06435	76.21453	86.49759
July	102.0911	106.5535	97.86288	103.471	49.21546	53.10025	86.43535	88.74331
August	100.6552	97.87422	89.43793	92.50319	91.15959	107.1625	98.04419	97.50161
September	119.887	116.5783	115.874	115.9565	178.7983	192.2215	86.8139	92.93531
October	145.8041	144.8916	105.9172	107.4228	349.1772	341.3856	118.2335	130.0645
November	104.2097	98.81823	99.71588	102.526	206.4294	194.3333	85.17352	82.64244
December	93.85225	86.53199	105.7443	102.4257	73.01952	69.97125	159.6215	110.0028
Grand average	708.0278	788.5972	674.5833	776.0093	217.75	236.7639	132.0833	148.4306

**Table 2:** Trend and MAPE of predictions obtained by ARIMA, Ratio-to-Trend, Simple Average and Simple average with mean annual increment (SAMAI) methods of Blood donation, Blood issue, RDP issue and FFP issue of data set I and II.

**Data sets**		**ARIMA**		**Ratio-to-Trend**		**Simple Average**	**SAMAI**	
		**Trend**	**MAPE (%)**	**Trend**	**MAPE (%)**	**MAPE (%)**	**Yearly average increase**	**MAPE (%)**
Blood Donation	I	decrease	12.99	decrease	21.08	20.05	1.092	18.82
	II	increase	13.51	increase	14.51	15.49	1.034	14.88
Blood issue	I	decrease	19.59	decrease	21.65	12.09	1.029	13.39
	II	decrease	21.6	decrease	14.52	18.18	0.989	17.23
RDP issue	I	increase	37.15	increase	25.62	29.06	1.388	14.52
	II	increase	27.35	increase	22.36	26.08	1.127	19.64
FFP issue	I	increase	31.94	decrease	39.95	34.85	1.111	27.64
	II	increase	23.78	increase	20.49	22.59	1.019	21.11
Average MAPE	I	-	25.42		27.08	24.01		18.59
Average MAPE	II	-	21.56		17.97	20.59		18.22
Average MAPE	I & II	-	23.49		22.52	22.3		18.4

**Table 3:** Seasonal index intercept and slope of Ratio-to-trend method for Blood donation, Blood issue, RDP issue and FFP issue for data set I and II.

	**Blood Donation**		**Blood issue**		**RDP issue**		**FFP issue**	
**Months**	**Data set I**	**Data set II**	**Data set I**	**Data set II**	**Data set I**	**Data set II**	**Data set I**	**Data set II**
January	87.47381	87.21667	91.153	92.944	33.964	31.409	113.647	115.491
February	98.41907	93.45956	96.257	92.387	35.352	27.479	89.213	91.119
March	100.8598	98.26992	104.949	100.947	52.366	43.43632	124.033	131.177
April	75.08248	77.52201	97.416	99.251	46.078	48.789	84.123	88.664
May	81.92207	85.81213	90.175	95.786	40.421	42.65	73.221	86.562
June	87.79534	95.13195	96.171	92.645	45.004	48.934	76.137	86.401
July	102.1911	108.5713	98.013	103.54	49.212	53.032	86.844	88.619
August	100.8216	100.0719	89.479	92.532	91.258	106.991	99.633	97.014
September	120.218	118.5092	117.729	116.229	178.988	192.303	86.999	92.829
October	145.984	147.0593	107.525	107.731	349.03	341.764	118.288	129.914
November	104.9129	100.1648	102.534	103.089	205.529	193.547	85.748	82.461
December	94.31975	88.21128	108.598	102.917	72.797	69.667	162.115	109.749
intercept	704.4	795.68	653.73	771.19	219.22	238.87	130.65	149
slope	-7.25	14.17	-41.71	-9.63	2.93	4.22	-2.86	1.15

**Table 4:** Auto-ARIMA results for data set I and II.

	**Blood Donation**		**Blood issue**		**RDP issue**		**FFP issue**	
	**Data set I**	**Data set II**	**Data set I**	**Data set II**	**Data set I**	**Data set II**	**Data set I**	**Data set II**
ADF-p	0.0002	0.818	0.1424	0	0	0.262271	0.0025	0.000018
Model	(1,0,0) (0,0,0)[12]	(0,0,0) (2,0,0)[12]	(0,1,1) (0,0,0)[12]	(0,0,0) (0,0,0)[12]	(0,0,1) (0,0,1)[12]	(0,0,2) (2,0,1)[12]	(1,0,1) (0,0,0)[12]	(0,0,0) (0,0,0)[12]
AIC	980.213	936.936	892.943	865.282	979.44	917.253	809.551	750.382
BIC	987.043	946.043	897.469	869.836	988.546	933.19	818.657	754.935
Model parameters	Coef ± std err	Coef ± std err	Coef ± std err	Coef ± std err	Coef ± std err	Coef ± std err	Coef ± std err	Coef ± std err
Intercept	308.2157 ± 79.659	224.5853 ±79.703	-	776.0093 ±11.346	211.4559 ±106.592	7.6286±8.419	34.3397±31.554	148.4306±6.344
ar.L1	0.5652 ± 0.078	-	-	-	-	-	0.7422±0.238	-
ar.S.L12	-	0.2556±0.233	-	-	-	0.2944±0.078	-	-
ar.S.L24	-	0.4598±0.197	-	-	-	0.6685±0.056	-	-
ma.L1	-	-	-0.5347±0.110	-	0.6043±0.122	0.4449±0.147	-0.4415±0.339	-
ma.L2	-	-	-	-	-	0.0549±0.330	-	-
ma.S.L12	-	.	-	-	0.3496±0.200	-0.4294±0.232	-	-
sigma2	44410 ±5376.09	20780 ± 2755.579	15970 ±2661.825	9176.29 ± 1185.866	41210 ± 5417.226	10180 ±1277.726	3990.489 ± 436.702	1860.387 ± 298.654

## References

[1] Uthayakumar R, Priyan S. (2013). Oper Res Health Care..

[2] Privett N, Gonsalvez D. (2014). Oper Res Health Care..

[3] Addis Bet (2015). Oper Res Health Care..

[4] Ben Elmir W (2023). Information..

[5] Fattahi M (2016). Ann Oper Res..

[6] Li Na (2021). Oper Res Health Care..

[7] Greinacher A (2007). Transfusion..

[8] Guo K (2022). Front Med (Lausanne)..

[9] Eichler H (2021). Transfus Med Hemother..

[10] Zeger SL (2006). Annu Rev Public Health..

[11] Huppert TJ (2009). Appl Opt..

[12] Jandoc R (2015). J Clin Epidemiol..

[13] Grzegorczyk M (2019). Methods Mol Biol..

[14] Lavrenz SM (2018). Accid Anal Prev..

[15] https://ijsrnsc.org/pdf_paper_view.php?paper_id=403&2-IJSRNSC-00581.pdf.

[16] Nandi AK (2020). Transfusion..

[17] Feng YN (2021). Mil Med Res..

[18] Feng Y (2021). Transfus Med..

[19] Liu S (2021). Ann Transl Med..

[20] Liu LP (2021). Front Med (Lausanne)..

[21] https://www.isroset.org/journal/IJSRBS/full_paper_view.php?paper_id=2438.

[22] Sun X (2021). Indian J Hematol Blood Transfus..

[23] https://www.egyankosh.ac.in/bitstream/123456789/20805/1/Unit-14.pdf.

[24] Saboia JLM. (1977). J Am StaAsso..

[25] Dickey D, Fuller W. (1979). J Am Stat Assoc..

[26] Dhara VR (2013). Indian J Med Res..

[27] Thakur SK (2023). Blood Sci..

